# Multicenter Evaluation of Two Next-Generation HIV-1 Quantitation Assays, Aptima Quant Dx and Cobas 6800, in Comparison to the RealTi*m*e HIV-1 Reference Assay

**DOI:** 10.1128/JCM.00292-18

**Published:** 2018-09-25

**Authors:** Frank Wiesmann, Robert Ehret, Gudrun Naeth, Martin Däumer, Jörg Fuhrmann, Rolf Kaiser, Christian Noah, Martin Obermeier, Gunnar Schalasta, Carsten Tiemann, Eva Wolf, Heribert Knechten, Patrick Braun

**Affiliations:** a PZB Aachen, Medical Center for Infectious Diseases, Aachen, Germany; b MVZmib AG, Medical Center for Infectious Diseases, Berlin, Germany; c Labor 28, Berlin, Germany; d University of Cologne, Institute of Virology, Cologne, Germany; e Labor Krone, Laboratory for Medical Diagnostics, Bad Salzuflen, Germany; f Labor Enders, Laboratory for Medical Diagnostics, Stuttgart, Germany; g IIG-Institute of Immunology and Genetics, Kaiserslautern, Germany; h MUC Research, Munich, Germany; i MVZ Labor Lademannbogen, Hamburg, Germany; Rhode Island Hospital

**Keywords:** Abbott, Aptima, Cobas 6800, HIV, Hologic, RNA, RealTi*m*e, Roche, viral load, viral load monitoring

## Abstract

High accuracy and precision at the lower end of quantification are crucial requirements of a modern HIV viral load (VL) assay, since some clinically relevant thresholds are located at 50 and 200 copies/ml. In this study, we compared the performance of two new fully automated HIV-1 VL assays, Aptima HIV-1 Quant Dx and Cobas HIV-1 (Cobas 6800), with the established RealTi*m*e *m*2000 assay. Assay precision and accuracy were evaluated in a retrospective evaluation out of excess plasma material from four HIV-1+ individuals (subtypes B, C, CRF01_AE, and CRF02_AG). Native plasma samples were diluted to nominal concentrations at 50 and 200 copies/ml (according to the RealTi*m*e *m*2000 assay). All dilutions were tested in triplicate in five independent runs over 5 days and in three labs per system. Assay concordance was determined using 1,011 surplus clinical routine samples, as well as selected retrospective longitudinal samples from 7 patients on treatment. The three assays yielded highly concordant results for individual clinical samples (*R*^2^ > 0.98; average difference, ≤0.2 log copies/ml) and retrospective longitudinal samples from patients on treatment. The Aptima and RealTi*m*e assays showed similar high precision, meeting the 5σ criterion for the majority of samples across all labs and subtypes. The Cobas assay was less precise, missing the 5σ criterion for the majority of samples at low concentrations. In this analysis, results from the Cobas assay appeared less reliable near the clinically relevant cutoff and should be interpreted with more caution in this context. Due to high precision, full automation, and high concordance with the RealTi*m*e assay, the Aptima assay represents a good alternative in routine VL monitoring.

## Abstract (precis)

High accuracy and precision at the lower end of quantification are crucial requirements of a modern HIV viral load (VL) assay, since some clinically relevant thresholds are located at 50 and 200 copies/ml. In this study, we compared the performance of two new fully automated HIV-1 VL assays, Aptima HIV-1 Quant Dx and Cobas HIV-1 (Cobas 6800), with the established RealTi*m*e *m*2000 assay.

## INTRODUCTION

The primary surrogate marker for the prediction of therapy success is the quantitation of HIV-1 RNA, or viral load (VL), in a patient's plasma (1–4). VL should be measured in all HIV-1-infected patients at entry into care, at initiation of therapy, and on a regular basis thereafter. Therapeutic success has been defined as the suppression of VL to <50 copies/ml both in clinical trials and in clinical routine (1–4). Nevertheless, due to a constant virus release out of the proviral CD4 T-cell reservoir, some treated patients still have detectable low-level plasma viremia above 50 copies/ml but below 200 copies/ml. Despite a well-functioning therapy in terms of improved immune parameters and the absence of disease progression and treatment adverse effects, as well as a low risk of emerging resistance, this release takes place (5). Thus, current therapy guidelines for HIV-1 have defined virologic failure as a VL repeatedly exceeding 200 copies/ml (2), which indicates that the current treatment has failed and needs to be reevaluated and probably substituted by an alternate treatment. Patients with VLs of 50 to 200 copies/ml, however, may continue with the same treatment and have their VL monitored more frequently (at least every 3 months) (2). There is no consensus on how to manage patients in this range. The risk of emerging resistance is thought to be low. It is suggested to confirm that levels remain above the lower limit of detection (LLOD) and to assess adherence and possible drug-drug or drug-food interactions.

A sudden rise in VL above the 200 copies/ml threshold may indicate an acute change in the patient's status (e.g., virologic failure) or may just be a viral “blip,” an isolated detectable VL observed after virologic suppression and subsequently followed by a return to virologic suppression or unconfirmed upon repeat testing (2). Blips are not associated with an increase in treatment failure and are generally considered artifacts due to random assay variation rather than clinically significant elevations in viremia (6, 7).

Thus, identification and confirmation of true HIV-1 virologic failure are critical for patient management, as patients typically visit physicians only twice per year in the United States or United Kingdom (4 times in Germany). Imprecision in a test result at low VLs (around the 50 and 200 copies/ml cutoffs) could have important medical implications, such as a patient remaining on a failing therapy, possibly leading to resistance, disease progression, and potential increase of transmission. Consequently, current HIV-1 VL assay systems need to deliver precise, reproducible, specific, and sensitive results, particularly around the clinical relevant benchmarks (50 and 200 copies/ml). Because patient management decisions rely entirely on each test result, the reliability of the HIV VL assay used in the clinical laboratory should be assessed and confirmed through objective rigorous modern analysis methods, such as the sigma metrics (8). The Greek letter sigma is used to represent standard deviations and to show how wide the distribution of values spans. Samples with a high standard deviation are more spread out and show more variability, while samples with a low standard deviation cluster more tightly around the corresponding mean. In quality control processes, it is important to know how many values (standard deviations) fit into a certain defined acceptable range (total analytical error). Therefore, it is recommended that laboratories estimate the total analytical error by combining the estimate of bias from a method comparison study and the estimate of precision from a replication study. Accordingly, we used a multiple of the standard deviation (SD) or coefficient of variation (CV) and total analytical error/bias for a 95% confidence interval, as recently published (8).

Currently, the most widely used HIV RNA quantitation assays in Germany are the Abbott *m*2000 RealTi*m*e HIV-1 (here, RealTi*m*e) and the Roche Cobas AmpliPrep/Cobas TaqMan HIV-1 v2 (here, CAP/CTM) assays, with lower limits of quantitation (LLOQs) of 40 and 20 copies/ml, respectively. The recent Hologic Aptima HIV-1 Quant Dx (here, Aptima) assay and the new Roche Cobas HIV-1 assay for use in the 6800/8800 systems (here, Cobas 6800/8800) are new adapted VL assays for the quantitation of HIV-1 on fully automated instrument platforms offering random access and LLOQs of 30 and 20 copies/ml, respectively. All comparative studies published to date have demonstrated that the Aptima assay is highly sensitive, precise, accurate, and highly concordant with other assays (9–19). Although there are currently no publications assessing the performance of the Cobas 6800/8800 HIV assay, two publications on the Cobas 6800/8800 system for human hepatitis C virus (HCV) VL determination have reported good analytical performance (20) and good correlation with the CAP/CTM v2 assay (21).

This systematic and comprehensive study had three aims. First, we compared the precision of the three assays around the clinically relevant cutoffs of 50 and 200 copies/ml in a multicenter approach using clinical samples from patients infected with the four most prevalent HIV-1 subtypes worldwide. A sigma metrics analysis was used to provide an objective evaluation of the assays' reliability. Second, we compared the results from the three assays in serial retrospective blood samples from patients receiving antiretroviral therapy over time. Third, we compared VL quantitation results from the Aptima, RealTi*m*e, and Cobas 6800 assays in a large number of prospective fresh clinical samples from HIV-infected patients.

## MATERIALS AND METHODS

### HIV-1 VL assays.

The Hologic Aptima HIV-1 Quant Dx assay was used on the fully automated Panther system (Hologic, Inc., San Diego, CA). The assay targets the polymerase (*pol*) and long terminal repeat (LTR) regions. The assay has a minimal required sample volume of 0.7 ml of specimen (0.5 ml plus 0.2 ml of dead volume) and reports quantitative HIV-1 results in a range of 30 to 10,000,000 copies/ml (22).

The Roche Cobas HIV-1 assay was performed on the fully automated 6800 system (Roche Molecular Diagnostics, Pleasanton, CA). The assay targets the *gag* gene and LTR region (dual target). The assay requires at least 0.655 ml of specimen (0.5 ml plus 0.15 ml of dead volume) and reports quantifiable HIV-1 results between 20 and 10,000,000 copies/ml (23).

The Abbott RealTi*m*e HIV-1 Quant Dx assay was used on the automated *m*2000 system (Abbott Molecular, Inc., Des Plaines, IL). The assay targets the *pol* integrase region (single target). The assay is designed to use 0.2, 0.5, 0.6 (used in this study), or 1.0 ml of specimen and reports quantifiable HIV-1 results over the range of 40 to 10,000,000 copies/ml (24).

### All three assay manufacturers report 100% specificity in the respective package inserts.

All laboratories used the same sample loading volume specified in the package insert when the same assay was tested at different sites and with different sample handling methods. Assays were performed in accordance to the instructions by trained technicians who had demonstrated proficiency. For laboratories using the newly introduced Aptima assay, at least one Panther operator per site was required to demonstrate assay proficiency prior to the study initiation. The proficiency evaluation involved testing Hologic panels, including a dedicated HIV proficiency panel.

### Assay precision analysis for different subtypes at low viremia in a multicentric approach.

**(i) Evaluation of assay precision**. Four clinical samples covering HIV-1 subtypes B, C, CRF01_AE, and CRF02_AG (1 sample each) were pretested with the RealTi*m*e assay and diluted in BaseMatrix to the nominal concentrations of 50 copies/ml and 200 copies/ml to test for subtype-specific assay precision at the lower end of quantitation. In order to minimize operator-related errors, triplicates of each dilution were tested in 5 independent runs with the Aptima, RealTi*m*e, and Cobas 6800 assays in nine laboratories, with 3 laboratories testing each assay. The same lot numbers were used for quantification processes as well as the same software versions on all systems used to minimize systematic errors. The three Aptima testing centers were PZB Aachen, Lab Enders, Stuttgart, and MIB, Berlin; the three RealTi*m*e testing centers were PZB Aachen, Labcon-OWL Analytik, Bad Salzuflen, and Institut für Immunologie und Genetik, Kaiserslautern; the three Cobas 6800 testing centers were Institut für Virologie, Koeln, MUC Research GmbH/MVZ Karlsplatz, Munich, and Labor Lademannbogen, Hamburg (all laboratories are in Germany). Samples were prepared in one laboratory (Aachen) and distributed frozen on dry ice to the testing laboratories. Each laboratory used an identical sample volume and identical sample handling in the testing process. The average assay values and 95% confidence interval (CI) were plotted by assay, subtype, and dilution to determine assay precision.

### (ii) RoU analysis.

An additional statistical analysis was conducted to evaluate the range of uncertainty (RoU) based on the precision and mean difference in assay values compared with the RealTi*m*e results. Calculations for the lower limit (equation 1) and upper limit (equation 2) of the RoU were derived from two one-sided confidence limit calculations approaching the clinical decision point from the lower or higher VL, as previously described (25):

(1) $${\text{RL}}_{\text{Low}}=\frac{{\text{CO}}_{\text{A}}}{1+\left(z\times \frac{\text{CV}\%}{\sqrt{n}}\right)}$$

(2) $${\text{RL}}_{\text{Up}}=\frac{{\text{CO}}_{\text{A}}}{1+\left(\frac{\text{CV}\%}{\sqrt{n}}\right)}$$

where RL is the limit of the range of uncertainty, CO_A_ is the assay-specific equivalent of the clinical cutoff in the reference assay, CV% is the assay-specific coefficient of variation at the respective cutoff level, and *n* is the number of replicates. Calculations were assuming a 95% confidence level, resulting in a *z* value of 1.645.

### (iii) Sigma analysis.

A sigma analysis of imprecision was also conducted, as described by Westgard and Lucic (8), for quality controls in which the sigma metric is calculated using the following equation: sigma metric = (TEa − bias)/%CV, where TEa is the allowable total error and CV is the coefficient of variation. The %CV values were calculated from nonlogarithmic values. The sigma metric was used to determine how likely it would be to identify a real change in the VL within the range of 50 to 200 copies/ml that represents the two clinically relevant thresholds (2). The sigma metric was graphed as a method decision chart that plots the %bias (using the assay-specific cutoff equivalent) versus the %CV. Statistical analysis was conducted by using SPSS version 21.0, Graph Pad Prism 5.0, and MedCalc version 17.4.4.

### Assay comparison in retrospective clinical samples at different time points during antiretroviral therapy.

In a retrospective evaluation, samples from 7 individual patients undergoing antiretroviral therapy were reanalyzed anonymously with the Aptima, RealTi*m*e, and Cobas 6800 assays by using leftover EDTA-plasma samples. For each patient, undiluted samples from five serial/consecutive time points during antiretroviral treatment were analyzed. The patients were selected to represent the majority of HIV group M subtypes (A, B, C, CRF02_AG, and CRF01_AE), to cover the dynamic quantitation range (from 30 to 10,000,000 copies/ml), based on the initial VL monitoring values measured with the RealTi*m*e assay, and to reflect different key steps and time points in the course of antiretroviral therapy, such as baseline VL, viral breakthrough, low-level viremia, and successful suppression of VL below the limit of quantification. Each assay was performed by one laboratory, as follows: Aptima testing and RealTi*m*e testing were performed at the PZB Aachen, Aachen, Germany, and Cobas 6800 testing was performed at the Institute of Virology at the University of Cologne, Germany.

### Assay comparison in fresh clinical samples.

Fresh consecutive clinical routine samples (*n* = 1,011) from HIV-1-infected patients seen for HIV-1 monitoring at the MIB Dienstleistung GmbH, Berlin, Germany, were tested fresh side by side with the routinely used RealTi*m*e assay and in parallel with the Aptima, RealTi*m*e, and Cobas 6800 assays during a period of 1 week (5 testing days). Plasma was obtained by centrifugation directly after arrival at the MIB laboratory. The only criterion for inclusion was sufficient plasma volume to test in all three assays. RealTi*m*e and Aptima testing was performed batch-wise at MIB, and Cobas 6800 testing was performed at Labor 28, Berlin. Samples were transported twice daily from MIB to Labor 28 in a frozen state to ensure real side-by-side comparison. All samples underwent the same freeze/thaw cycle and treatment conditions. The results were grouped in three categories, as follows: “not detected,” “detected <LLOQ,” and “quantitated” using the individual assays' LLOQs (RealTi*m*e assay, 40 copies/ml; Aptima, 30 copies/ml; and Cobas 6800, 20 copies/ml). Assay results were also compared at the clinically defined cutoff of 50 copies/ml and at low VL (between 50 and 250 copies/ml) using Venn diagrams. Agreement between assays for the 50 copies/ml cutoff was determined using kappa statistics. The overall concordance of VL values for samples quantitated in all three assays was analyzed pairwise by Deming regression (with calculation of the correlation coefficient *R*) and by Bland-Altman analysis (with calculation of the mean difference and the 95% assay agreement).

## RESULTS

### Assay accuracy and precision.

**FIG 1:**
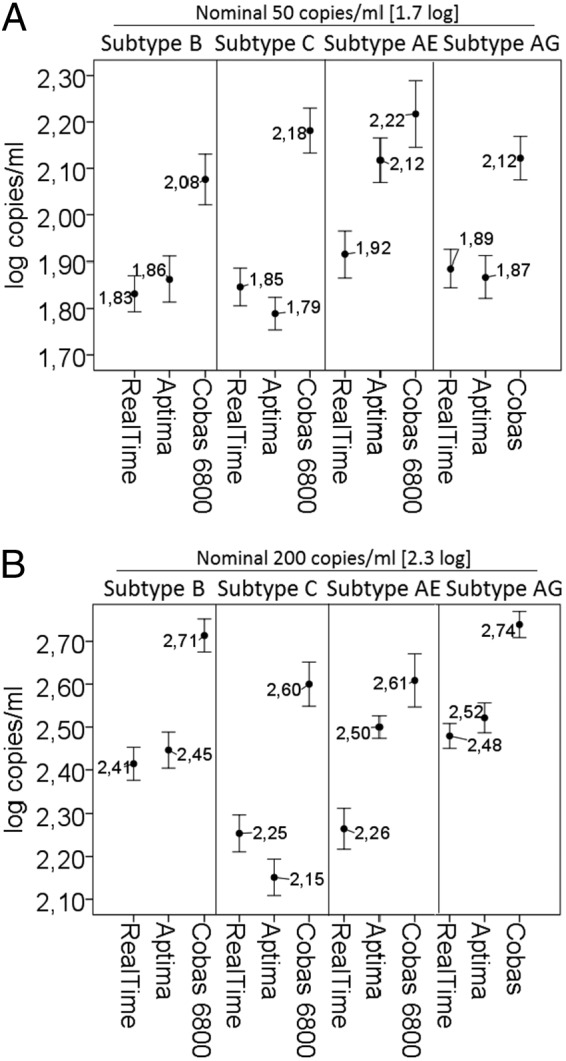
Assay accuracy and precision with various subtypes for replicates at 50 copies/ml (A) and 200 copies/ml (B). Whiskers plot with center dots represent the mean value (which is indicated in each box) and bars represent the 95% CI.

The RealTi*m*e and Aptima assays both yielded results closest to the target values (within 0.13 and 0.20 log/copies/ml, respectively), while the Cobas 6800 assay was the least accurate, yielding results on average 0.41 log copies/ml from the target values (RealTi*m*e), with systematically higher confidence intervals (95%) across all subtypes (Fig. 1). For the 50 copies/ml replicates, the majority (≥95%) of the samples would be expected to be located below the clinically relevant cutoff of 200 copies/ml for all three assays (Fig. 1a). For the replicates with 200 copies/ml, the Cobas 6800 assay systematically yielded values clearly above this cutoff, while the RealTi*m*e and Aptima assays yielded results both above and below the 200 copies/ml cutoff (Fig. 1b).

Of all three assays, the Cobas 6800 had the lowest precision for all subtypes and VL levels (50 and 200 copies/ml), as shown by the largest 95% CIs and coefficients of variation for the 45 replicates tested (Fig. 1).

In general, the best precision of all assays was achieved for subtypes B and CRF02_AG. At the critical 50 copies/ml threshold, the Aptima and RealTi*m*e assays performed equally well for these subtypes, with an SD ≤0.15 log. At this level, a higher imprecision was observed with the Cobas 6800 assay and subtype CRF01_AE, with an SD of 0.23 log.

### Analysis of range of uncertainty.

**FIG 2:**
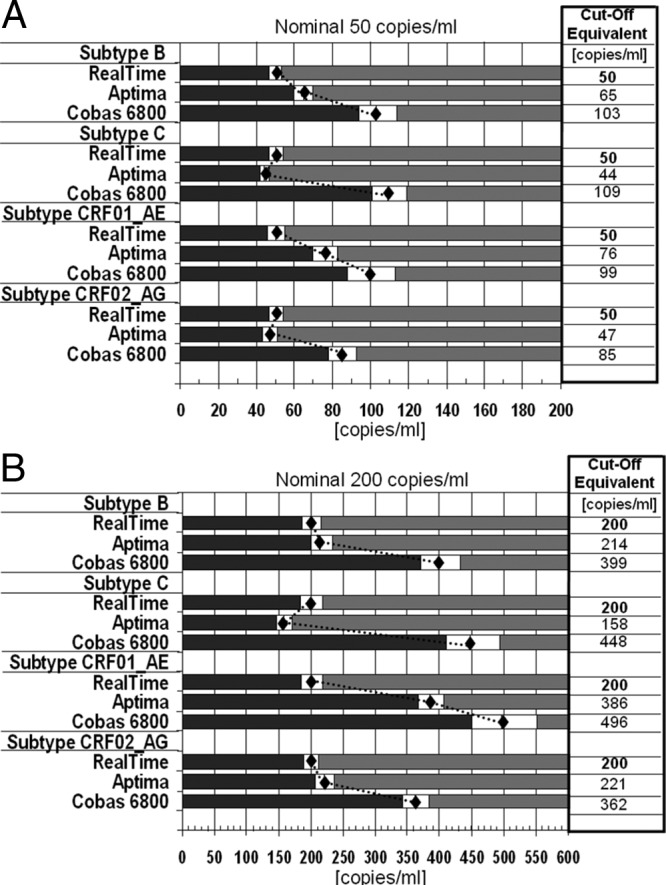
RoU for replicates at 50 copies/ml (A) and 200 copies/ml (B) in relation to the RealTi*m*e assay (reference). Gray bars represent areas of ≥95% confidence that do not cross the decision threshold of 50 or 200 copies/ml for the reference assay (RealTi*m*e). Diamonds represent the assay-specific values corresponding to the reference assay threshold value. White bars around the diamond represent the RoU of each assay's results. COa, assay-specific equivalent of the clinical cutoff in the reference assay (Abbott *m*2000 RealTi*m*e).

RoU analysis confirmed these results. Of all three assays, the Cobas 6800 assay had the lowest precision for all subtypes tested and VL levels (50 and 200 copies/ml), as shown by the widest RoU and values much higher than those of the other two assays (Fig. 2). As an example, for subtype C replicates, a 100 copies/ml result in the Cobas 6800 assay would correspond to <50 copies/ml with the RealTi*m*e and Aptima assays in ≥95% of the replicates. The Aptima results were in close agreement with the RealTi*m*e results for 3 out of the 4 subtypes tested (B, C, and CRF02_AG) (Fig. 2). The Aptima assay showed the highest precision (smallest RoU) with subtype C, while the RealTi*m*e assay showed highest precision with subtypes B, CRF01_AE, and CRF02_AG, with only slight differences from the Aptima assay for subtypes B and CRF02_AG (Fig. 2).

### Differences in results between laboratories.

Significant interlaboratory differences were observed with the RealTi*m*e assay for subtypes B, C, and CRF02_AG for the 200 copies/ml replicates and with the Cobas 6800 assay for subtypes C and CRF01_AE for all VLs tested (50 and 200 copies/ml), while none were observed for the Aptima assay (Table 1). In fact, we found that approximately half of the 50 copies/ml replicates of subtype CRF01_AE were quantitated above the cutoff of 200 copies/ml when tested with the Cobas 6800 assay in laboratory 3, while all samples were quantified below this cutoff when being tested in laboratory 2. A similar systematic error was observed for the subtype C results. In contrast, the majority of subtype replicates were quantified below 200 copies/ml with the Aptima and RealTi*m*e assays.

**TABLE 1:** Test for significant differences between results obtained from the three different participating laboratories

Subtype	Nominal concn (copies/ml)^*a*^	ANOVA *P* value by assay^*b*^		
		RealTi*m*e	Aptima	Cobas 6800
B	50	0.014	0.750	0.218
	200	**0.005**	0.517	0.357
C	50	0.113	0.580	**<0.001**
	200	**0.003**	0.581	**<0.001**
CRF01_AE	50	0.351	0.226	**<0.001**
	200	0.041	0.015	**<0.001**
CRF02_AG	50	0.726	0.103	0.462
	200	**<0.001**	0.404	0.082

a Determined by *m*2000 RealTi*m*e HIV-1 assay.

b Values indicated in bold are statistically significant at a *P* value of <0.01. ANOVA, analysis of variance.

### 6σ analysis of imprecision.

**FIG 3:**
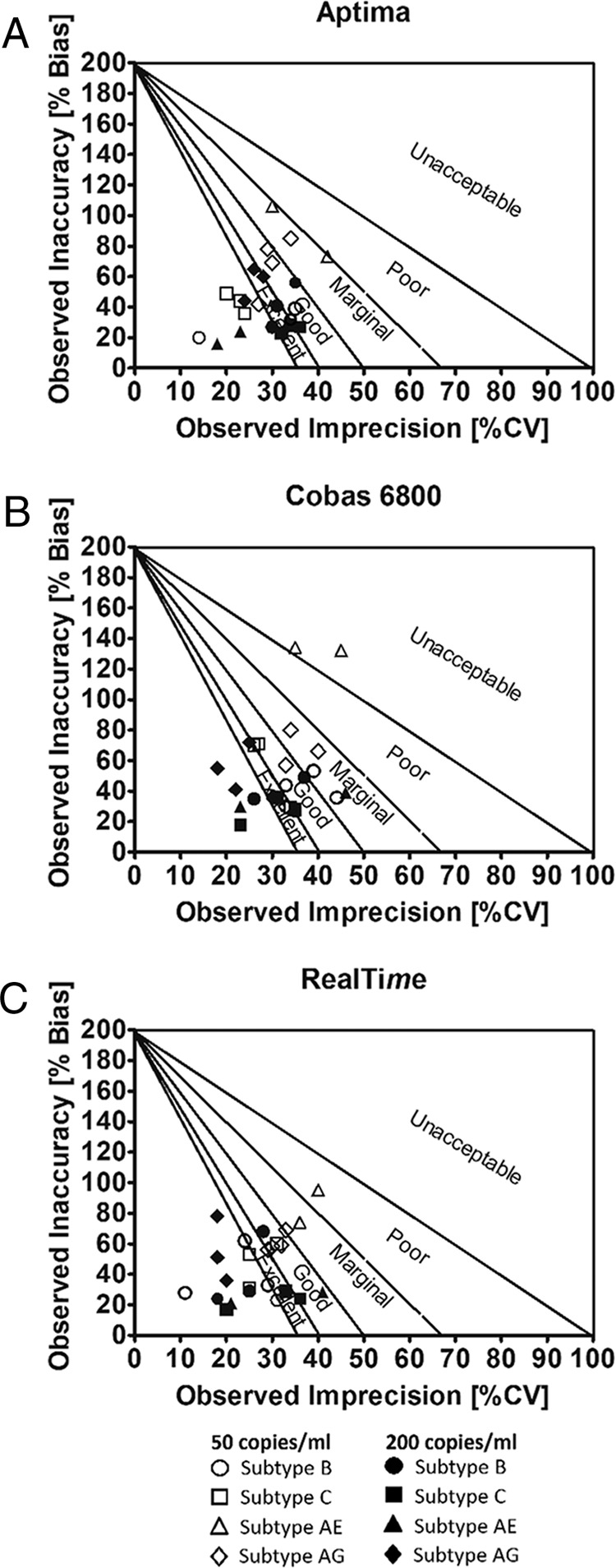
Sigma precision analysis for all laboratories and all assays. Plots were performed according to Westgard and Lucic (8) for each participating laboratory, subtype, and dilution. Diagonal lines on the chart represent different sigma zones, from world-class-quality performance (more than 6σ) near the graph's origin, to excellent performance (5σ), good performance (4σ), etc., with the upper right quadrant of the graph representing less than 2σ performance, which is considered unacceptable, unstable, and unreliable. Each symbol corresponds to one subtype measured in three different labs. The open symbols represent 50 copies/ml, and the filled symbols represent 200 copies/ml.

Aptima was the only assay exceeding the 3σ criterion for all subtypes at all VLs tested (50 and 200 copies/ml) (Fig. 3). The RealTi*m*e assay failed for subtype CRF01_AE replicates at 50 copies/ml. However, the majority of the Aptima and RealTi*m*e results exceeded the 5σ criterion for excellence. The majority of the Cobas 6800 results did not reach the 5σ level, particularly for replicates at 50 copies/ml. Subtype CRF01_AE results failed to pass the 1σ criterion in this analysis.

### Longitudinal patient VL monitoring.

**FIG 4:**
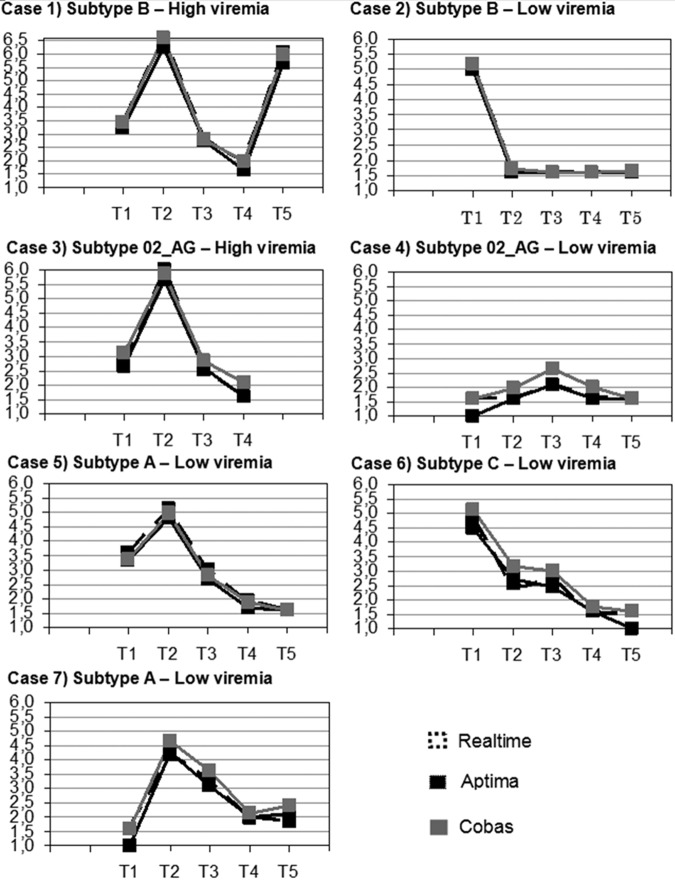
Longitudinal HIV-1 RNA monitoring in 7 patients (2 subtypes B, 5 non-B). T1 to T5 represent 5 different consecutive time points, with corresponding logarithmic viral loads on the *y* axis. Black, Aptima results; dashed lines, RealTi*m*e results; gray, Cobas 6800 results.

Serial retrospective samples from 7 patients (two subtypes B, two CRF02_AG, one CRF01_AE, one A, and one C) receiving different courses of therapy showed an excellent agreement between all three assays across a wide range of VLs, different HIV subtypes, and therapy regimens (Fig. 4). Generally, the Aptima results tended to be close to the RealTi*m*e values, with both assays slightly below the corresponding Cobas 6800 values. The lowest average difference was observed between the Aptima and RealTi*m*e (0.13 log copies/ml) results. There was a trend for the Cobas 6800 assay to return slightly higher values across the time course and more noticeably at low viral loads, except for subtype A. The average values for the Cobas 6800 assay were 0.17 and 0.30 log_10_ copies/ml higher than those for the Aptima and RealTi*m*e assays, respectively.

### Concordance of clinical sample results in the three assays.

(i) Concordance of qualitative results. A total of 1,011 fresh clinical leftover samples were analyzed side by side in the three assays. Concordance between the three assays for characterizing the samples' VLs as detected, detected ≤LLOQ, or quantitated differed depending on the cutoff chosen. Using the system-specific cutoffs, nearly twice as many quantitated results were reported with the Cobas 6800 assay than with the Aptima and RealTi*m*e assays (234 versus 140 and 136 samples, respectively; Table 2). Not detected results were highest with the RealTi*m*e assay (712 samples) and comparably lower with the Aptima (512 samples) and Cobas 6800 (506 samples) assays, respectively (Table 2), indicating a higher clinical sensitivity of the Aptima and Cobas 6800 assays.

**TABLE 2:** Characterization of sample detection by each assay

Assay result	No. of samples by assay (*n* = 1,011)^*a*^		
	RealTi*m*e	Aptima	Cobas 6800
Not detected	712	512	506
Detected <LLOQ	163	359	271
Quantitated	136	140	234

a LLOQ, lower limit of quantitation (RealTi*m*e, 40 copies/ml; Aptima, 30 copies/ml; Cobas 6800, 20 copies/ml).

**FIG 5:**
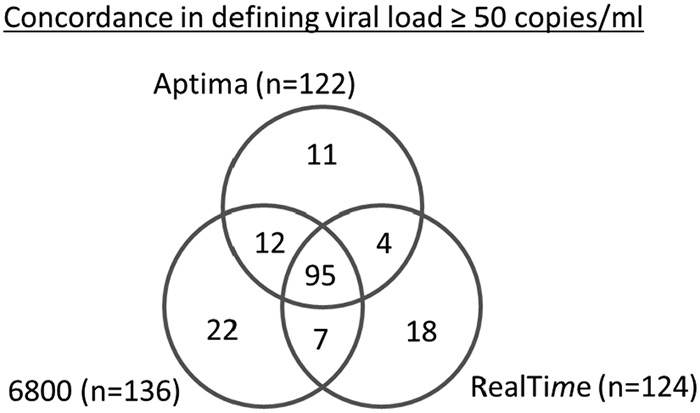
Assay concordance for quantitating samples at ≥50 copies/ml. Venn diagram indicates the number of samples quantitated at ≥50 copies/ml by one, two, or three of the assays. 6800, Cobas 6800.

Using the clinically established cutoff of 50 copies/ml as a benchmark reduced the differences between the assay results, with 122 (12.1%), 136 (13.5%), and 124 (12.3%) samples yielding quantitated results beyond 50 copies/ml with the Aptima, Cobas 6800, and RealTi*m*e assays, respectively (Table 3); in that case, the Cobas assay quantitated ∼10 to 12% more samples at >50 copies/ml than the other two assays. Of the samples quantitated at ≥50 copies/ml by each assay (122 samples with Aptima, 136 samples with Cobas 6800, and 124 samples with RealTi*m*e assays; Table 2), the Aptima assay showed the lowest number of results of >50 copies/ml not confirmed by any of the comparator assays (12), followed by the RealTi*m*e (19) and then Cobas 6800 (26) assays (Fig. 5). The overall agreement between assays for the quantitation of samples at the threshold of 50 copies/ml was high, as demonstrated by a kappa factor of 0.80 for the Aptima/Cobas 6800 comparison, 0.78 for the Aptima/RealTi*m*e comparison, and 0.75 for the RealTi*m*e/Cobas 6800 comparison, indicating almost perfect agreement in all cases.

**TABLE 3:** Characterization of sample concentrations by each assay

Assay result	No. of samples by assay (*n* = 1,011)		
	RealTi*m*e	Aptima	Cobas 6800
Not detected	712	512	506
Detected ≤50 copies/ml	175	377	369
Quantitated >50 copies/ml	124	122	136

Overall, 58, 72, and 65 samples were quantitated between 50 and 250 copies/ml by the Aptima, Cobas 6800, and RealTi*m*e assays, respectively. Thirty-nine samples were quantitated between 50 and 250 copies/ml by Aptima and Cobas 6800, 37 by Cobas 6800 and RealTi*m*e, 34 by Aptima and RealTi*m*e, and 26 by all three assays. The Aptima assay demonstrated the highest clinical sensitivity together with Roche and at the same time the lowest number of >50 copies/ml results not confirmed by any of the two comparator assays.

### (ii) Correlation of quantitative values.

**FIG 6:**
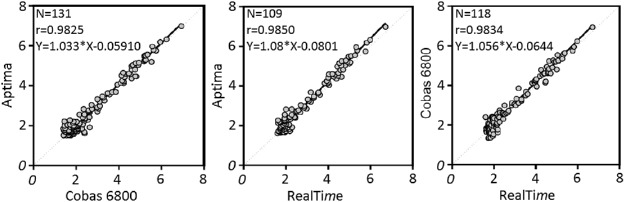
Deming regression analyses for assay comparisons. The solid lines represent assay results best fit. The dots represent logarithmic values of each tested clinical sample from one assay (*x* axis) versus the comparator assay (*y* axis).

In Deming regression analyses comparing the assays' VL values in a pairwise fashion, all comparisons led to *R* values above 0.98 (Fig. 6). Bland-Altman analyses showed that the average differences between paired assay results were all under 0.2 log copies/ml (Cobas 6800-Aptima = 0.038 log copies/ml; Cobas 6800-RealTi*m*e = 0.103 log copies/ml; Aptima-RealTi*m*e = 0.168 log copies/ml) and that >92% of the observed values were within the 95% confidence limits of agreements between the assays in all comparisons (data not shown).

## DISCUSSION

Correct quantitation of HIV-1 VL around 50 copies/ml (virologic suppression thresholds are generally regarded as below the limit of quantitation of current assays, 20 to 40 copies/ml) and 200 copies/ml (virologic failure threshold according to recent guidelines) during therapy is critical for making an adequate treatment decision in the management of HIV-infected patients. Indeed, quantifiable results above 200 copies/ml, which are suggestive of therapeutic failure or viral breakthrough, require repeat testing and, if confirmed, should prompt treatment modification. VL assays that have substantial variability around these cutoffs can yield false positives, which require repeat testing, or yield false negatives, masking true virologic failure, which leads to the patient remaining on an ineffective treatment. False positives have direct consequences on the patient's health, as they increase the risks of drug resistance, virus transmission, and clinical progression. It has been reported that although about 90% of the first-line-treated patients show suppressed viral loads below the quantification limits, another 3 to 4% are likely to remain somewhere between this critical range of 50 to 500 copies/ml (as tested with the RealTi*m*e assay) (27). Approximately 4% to 8% of the total population that achieved an initial plasma viral load below 50 copies/ml in the studies subsequently developed persistent low-level viremia of <500 copies/ml (28). Therefore, we would estimate the relative impact of quantifiable results in daily routine and within this critical <2.5-log range to be somewhere in the low-single-digit-percentage range. Thus, it is imperative that in order to be used for patient treatment monitoring, VL assays have great precision and accuracy (low noise level) at the low end of viral concentration (the 50 and 200 copies/ml cutoffs). Assays that can achieve 6σ performance at those cutoffs will by their nature have fewer false positives and false negatives, thereby reducing unnecessary repeat testing and avoiding medical errors.

Published reports have shown that the widely used CAP/CTM and RealTi*m*e assays may yield unexplained viral blips with no obvious clinical cause in patients on stable antiretroviral (ARV) therapy (7, 29, 30). It is possible that these blips are due to the viral load assay used and the underlying technology that may lead to the detection of proviral DNA (6). It has also been demonstrated that probes within the 2-LTR target regions may be more prone to result in higher coefficients of variation than probes within the *pol* region (31). Therefore, we aimed to compare the performances at the point of decision of two new fully automated VL assays for HIV quantitation, the Cobas 6800 and Aptima assays, with the conventional RealTi*m*e assay. Assay precision and accuracy were compared in replicates of clinical samples of various subtypes at the two clinically relevant thresholds of 50 and 200 copies/ml, respectively. Assay performance comparisons were performed in retrospective follow-up samples from 7 patients under antiretroviral treatment representing various HIV-1 subtypes and in 1,011 fresh clinical samples.

Accuracy and precision analyses at the two relevant cutoffs (50 and 200 copies/ml) showed the best accuracy and precision for the RealTi*m*e and Aptima results, with the two assays performing similarly, while the Cobas 6800 assay appeared to be more inaccurate. Indeed, the RealTi*m*e and Aptima assays yielded values that were within 0.20 log copies/ml from the target values, while the Cobas 6800 results were on average ∼0.4 log copies/ml systematically above the target. The Cobas 6800 assay was also much less precise than the RealTi*m*e and Aptima assays, as shown by a much wider RoU around the average values. Sigma analysis of assay accuracy and precision showed that, similar to RealTi*m*e, the Aptima assay was highly precise, meeting at least the 3σ requirement for all subtypes and viral load levels. Although Cobas 6800 performance reached 5σ (excellent) for the majority of higher-viremia replicates, its performance dropped down to 4σ and less for lower-viremia replicates.

As HIV assays exhibit various degree of variability at low VLs around the clinically relevant cutoffs, assay performance at these key thresholds can be objectively evaluated using the sigma metrics (8). Westgard and Lucic (8) used previously published data on HIV VL assays (6, 26) to retrospectively calculate the sigma metrics for two comparator assays and found the RealTi*m*e assay to be 6σ (world-class; in both studies) and the CAP/CTM assay to be 4σ (good in reference 6) to 3σ (marginal in reference 26). The Westgard sigma analysis for RealTi*m*e is in agreement with our findings.

In 1,011 fresh plasma samples, a qualitative comparison (quantitated versus not quantitated) of assay results using the assay-specific cutoffs showed that the Cobas 6800 assay quantitated nearly twice as many plasma samples than the Aptima and RealTi*m*e assays. This may be attributed to a combination of both amplification of cell-associated proviral DNA and increased detection of very low viral loads, as suggested by Ouma et al. (32). The first is relatively unlikely because the EDTA-blood samples were centrifuged immediately after entering the laboratory, and fresh plasma was used for all three assays. The RealTi*m*e and Cobas 6800 assays should at least be able to detect free plasma viral DNA in a comparable manner. The second seems unlikely because the limits of detection (LOD) for Cobas 6800 and Aptima are comparably low. Switching to the clinical validated cutoff of 50 copies/ml, the number of quantitated samples by the Cobas 6800 was ∼10 to 12% higher than with the Aptima and RealTi*m*e assays. In analyses of all quantitated results, the three assays showed high correlation of VL values, with *R*^2^ > 0.98 and average differences of <0.2 log copies/ml. These findings are comparable with those from prior studies that compared the Aptima and RealTi*m*e assays (13–15, 18, 19).

For longitudinal data from 7 patients under treatment, there was a high agreement with all three assays across a wide range of VLs and different HIV subtypes. Generally, the Aptima and RealTi*m*e assay values were closer, and slightly below the Cobas 6800 values, except in one patient (subtype A).

In conclusion, this study is the first comparing the Aptima, Cobas 6800, and RealTi*m*e assays for HIV VL quantitation side by side, and to use the sigma metrics in a primary analysis to objectively rate the assays' performance based on a multicenter evaluation. The RealTi*m*e and Aptima assays demonstrated a very high agreement, and the two assays performed equally well in relation to clinically relevant cutoffs, showing excellent accuracy and precision. In this analysis, the results from the Cobas 6800 assay appeared to be more discordant with the other two assays and less precise and accurate at the clinically relevant cutoffs (50 and 200 copies/ml). Thus, the Cobas 6800 results should be interpreted with more caution before clinical decisions are made. Of the three tested assays, Aptima was the only assay showing at least 3σ performance for all tested subtypes and dilutions. Assays that can achieve high σ performance inherently have fewer false positives and false negatives, which allows clinicians to unequivocally trust the assay results. Owing to its σ performance, high concordance with the RealTi*m*e reference assay, and user-friendly handling, full automation, flexibility of operation (random access), and efficient workflow (no batches), the Aptima assay represents a good choice for diagnostic laboratories performing routine monitoring of HIV-1 VLs.
